# Molecular markers associated with resistance to squash leaf curl China virus and tomato leaf curl New Delhi virus in tropical pumpkin (*Cucurbita moschata* Duchesne ex Poir.) breeding line AVPU1426

**DOI:** 10.1038/s41598-024-57348-9

**Published:** 2024-03-21

**Authors:** Roland Schafleitner, Lin Chen-yu, Suwannee Laenoi, Huang Shu-mei, Supornpun Srimat, Lee Gi-An, Orawan Chatchawankanphanich, Narinder P. S. Dhillon

**Affiliations:** 1 World Vegetable Center, 60 Yi-Min Liao, Shanhua, 74151 Tainan Taiwan; 2 World Vegetable Center, East and Southeast Asia, Kasetsart University, Kamphaeng Saen, Nakhon Pathom, 73140 Thailand; 3 National Agrobiodiversity Center, National Institute of Agricultural Sciences, Rural Development Administration, Jeonju, 54874 Republic of Korea; 4 National Center for Genetic Engineering and Biotechnology (BIOTEC), National Science and Technology Development Agency (NSTDA), Pathum Thani, 12120 Thailand

**Keywords:** Genetics, Plant sciences

## Abstract

Virus diseases are a major production constraint for pumpkin. Recessive resistance to squash leaf curl China virus and tomato leaf curl New Delhi virus has been mapped in *Cucurbita moschata* (Duchesne ex Poir.) breeding line AVPU1426 to chromosomes 7 and 8, respectively. Molecular markers tightly associated with the resistance loci have been developed and were able to correctly predict resistance and susceptibility with an accuracy of 99% for squash leaf curl China virus resistance and 94.34% for tomato leaf curl New Delhi virus in F_2_ and back cross populations derived from the original resistance source AVPU1426. The markers associated with resistance are recommended for use in marker-assisted breeding.

## Introduction

Pumpkin (*Cucurbita* spp.) comprises a variety of different species including *Cucurbita moschata, C. pepo, C. maxima, C. mixta, C. facifola* and *Telfairia occidentalis*^1^. *Cucurbita moschata* (tropical pumpkin) is the third most economically important *Cucurbita* species after *C. pepo* and *C. maxima*^2^. Pumpkin is a vegetable crop with high nutritional value, containing a wide range of essential macro- and micro-nutrients required in human diets, including vitamins, antioxidants and bioactive compounds^3^. Besides the flesh, also the peel, seeds or shoots of some pumpkin varieties are consumed. The flesh is a good source of β-carotene, and pumpkin seeds are rich in protein with a balanced amino acid content and low antinutrient levels such as phytic acids and trypsin inhibitor activity^4^, containing high amounts of B-complex vitamins and minerals such as Mg, Fe, Zn, P, K, Se, Mn and Cu.

Close to 28 million tonnes of pumpkins, squashes and gourds were harvested in 2020 from about 2 million ha worldwide^5^. Tropical pumpkin varieties (*C. moschata*) have a relatively short crop duration of 100–135 days and a yield potential between 15 and 30 t per ha. One of the benefits of pumpkin is that the mature fruit can be stored for extended periods, making the crop highly suitable for markets in low-income countries where cold storage facilities are lacking. The high yield potential and good storability, together with its high adaptability and nutritional quality, makes pumpkin a strategic crop for biofortification programs to ensure food and nutrition security^6,7^.

Virus diseases mainly caused by *Begomovirus* and *Potyvirus* species, several fungal diseases such as powdery and downy mildew, and insects pests, are the major biotic stress factors limiting pumpkin production and productivity^8–10^. Among the viruses, zucchini yellow mosaic virus (ZYMV), papaya ring spot virus (PRSV), watermelon mosaic virus (WMV), cucumber mosaic virus (CMV), squash leaf curl virus (SLCV), tomato leaf curl New Delhi virus (ToLCNDV) and cucurbit aphid borne yellow virus (CABYV) are the major constraints to pumpkin cultivation^8,11–13^. Resistance genes against *Potyvirus* species^14^, CMV (genus *Cucumovirus*^15^*,* and *Begomovirus*^16^) have been described, but resistance genes against CABYV *(Polerovirus)* have not yet been reported.

Diseases caused by *Begomovirus* species have become increasingly devastating for vegetable production^17^. The *Begomovirus* species ToLCNDV is present in Asia and in the Mediterranean on a wide range of host species^18^. It is transmitted by whiteflies, but can also be mechanically transmitted on melon^19^, and is seed borne in zucchini^20^ and chayote^21^. Leaf curl disease affected pumpkin leaves sampled in India that tested positive for ToLCNDV^22^. Infection symptoms included leaf curling, vein thickening, darker leaf margins, leaf area reduction, short internodes, and stunted plants^18^. Fruit cracking appears upon ToLCNDV infection in melon^23^. The bipartite genome of the virus consists of DNA-A and DNA-B components, which often interact with beta satellites that enhance the virulence of the virus^18,24^. Squash leaf curl disease caused by the begomovirus SLCV is among the most destructive diseases of cucurbit crops in tropical, subtropical and semiarid regions. This virus is prevalent in the Americas infecting cucurbits and some legumes^25^, and it was also reported on cucurbits in the Philippines^26^. Disease symptoms include severe stunting and leaf curling, resulting in major yield losses^27^. Squash leaf curl China virus (SLCCNV), a bipartite begomovirus, has been isolated from different cucurbit crops in Asia^28,29^, and SLC Philippine virus Taiwan strain (SLCPV-TW), also a bipartite begomovirus, was first isolated from pumpkin in Taiwan^30^.

Conventional begomovirus control applies whitefly management through pesticides, biocontrol, and physical barriers^31^. Early sowing, weed management, crop rotation, and roughing of infected plants can reduce disease incidence, but the most effective protection against yield losses from begomovirus infection is the development of virus and/or whitefly resistant varieties. A recessive gene conditioning ToLCNDV resistance was mapped in two *C. moschata* landraces PI 604,506 and PI 381,814 to chromosome 8^16^, while a dominant gene governing resistance to ToLCNDV in *C. moschata* accession BSUAL-252 originating from Japan was identified at a different genomic location^32^. A field screening effort of 800 *C. moschata* accessions identified ToLCNDV and SLCCNV resistant plants with desirable fruit traits. After hand self-pollination of resistant plants and subsequent pedigree selection, breeding line AVPU1426 with field resistance to SLCCNV and ToLCNDV was developed from the heterozygous and heterogeneous WorldVeg genebank accession VI056782 originally collected from Bangladesh, as a fourth resistance source to reduce begomovirus diseases on pumpkin. Resistance of AVPU1426 by was confirmed by testing the response to local isolates of SLCCNV and ToLCNDV in Thailand^33^. Resistance assessment in the F_1_ and in segregating F_2_ and backcross populations derived from a cross between AVPU1426 (resistant) and Waltham Butternut (susceptible), suggested that resistance to ToLCNDV and SLCCNV is governed by a single recessive gene^33^. However, it was not clear whether the same gene controls resistance to both viruses, or different recessive genes are implicated with resistance to each of the viruses. Furthermore, it was unclear whether the gene(s) controlling resistance against both viruses is/are different from the one previously mapped to chromosome 8 by^16^.

Marker-assisted selection has become a standard method in plant breeding. Markers associated with virus resistance make introgression of resistance genes into elite lines more effective. In particular, introgression of recessive resistance genes from non-adapted materials into elite lines through backcrossing is strongly facilitated by molecular markers, enabling foreground selection for individuals that are heterozygous for the resistance gene, and for background selection to restore the recurrent genotype in the population in as few generations as possible. For foreground selection, markers tightly linked with the gene of interest are required.

The present study describes the mapping of ToLCNDV and SLCCNV resistance genes in segregating pumpkin populations, and the validation of the obtained markers that are proposed for marker-assisted selection of the resistance genes provided by breeding line AVPU1426.

## Materials and methods

### Plant materials and virus resistance screening

From a cross between Waltham Butternut (susceptible) as the maternal line and AVPU1426 (resistant) as the paternal line, F_2_ and two backcross populations, one backcrossed to the susceptible and one to the resistant parent were produced^33^. Whitefly rearing, production of viruliferous whiteflies, experimental design, plant growth and the two experiments exposing the parental lines, the F_1_, the F_2_ and backcross populations to one of the two viruses ToLCNDV and SLCCNV were described by^33^. Seed of the experimental plants to assess the response to SLCCNV were sown on 20 September 2019. DNA was extracted from young leaves 11 days after sowing (DAS) using DNeasy Plant Mini Kit (Qiagen) according to the instructions of the supplier. Virus inoculation through exposure to viruliferous whiteflies was initiated 12 DAS. After 48 h the whiteflies were eliminated with insecticide and the plants were transplanted 17 DAS to 45 cm pots and kept in the greenhouse with virus symptoms scored 46 DAS, using a visual symptom rating scale as described in^33^: 0 = no visible symptom; 1 = mild symptom with very slight yellowing of leaves; 2 = moderate symptom with yellowing and mottling; 3 = severe symptom with wide range of leaf yellowing, curling and mottling; 4 = severe symptom with stunting of the plant, leaf yellowing, curling, and reduction of leaf lamina. Plant ratings 0–1 were considered resistant, whereas ratings 2–4 were considered susceptible^33^. For ToLCNDV resistance screening, seedlings were sown on 21 February 2020, DNA was extracted 11 DAS, virus inoculation through viruliferous whiteflies started 12 DAS, whitefly elimination and transplanting were performed as described for SLCCNV, and virus symptoms scoring and rating were carried out 42 DAS.

All plants used in the study, including the collection of the plant material, complied with relevant institutional, national and international guidelines and legislation. The virus resistant breeding line AVPU1426 is available from the World Vegetable Center upon request (https://avrdc.org/seed/seeds/).

### Genotyping-by-sequencing

DNA samples of 6 individuals of each parental line and of 82 F_2_ plants of the plants exposed to SLCCNV, and 4 plants of each parental line and 82 F_2_ plants of the plants exposed to ToLCNDV, were prepared for genotyping by sequencing. DNA concentration was standardized to 5ng/µL using Qubit. Sequencing libraries (ApeKI) were prepared according to the GBS protocol as per^34^, except for using centrifugal evaporation to enrich the PCR products of the library, which were subsequently separated on a 6% acrylamide gel. Gel pieces containing DNA fragments of a size between 300 and 400 bp were cut out, the DNA was recovered from the gel following^35^ and purified using the MinElute PCR fragment purification kit. The recovered DNA was again quantified (Qubit) and the samples derived from the SLCCNV infected plants were sent to the High Throughput Genomics Core Facility, Biodiversity Research Center, Academia Sinica, Taipei, Taiwan, for quality control on an Agilent DNF-474 HS NGS fragment analyzer and for sequencing (151 bp single reads) on a single flow cell of an Illumina HiSeq 2500 device. The samples from ToLCNDV were sent to Novogene for paired end (2 × 150 bp) sequencing on an Illumina HiSeq_X device. FastQ files were obtained from the sequencing providers. SNP calling in the SLCCNV experiment was performed in Tassel 5.0^36^ with the *Cucurbita moschata* (Rifu) whole genome sequence as a reference (http://cucurbitgenomics.org) using the Burrow-Wheeler Aligner bwa.07.17 for short-read alignment^37^. SNPs of the progenies were filtered for minimal sequencing depth (2) and maximum missing data (50%), except for chromosome 5, where a maximum missing data rate of 40% was allowed. Missing genotypes were imputed after removal of unmapped reads using FSFHap in Tassel using a window size of 50 and a window overlap of 25. The resultant SNPs were filtered for 20% minimum allele frequency and a maximum of 70% heterozygosity. Minor SNP states and sites with indels were removed.

For ToLCNDV, SNP calling in the parents and progenies was performed in Stacks v2.60^38^ also using the *Cucurbita moschata* (Rifu) whole genome sequence as a reference (http://cucurbitgenomics.org). SNPs were filtered for minimum sequencing depth of 4, maximum missing data of 50%, and minimum allele frequency of 10%. As above, minor SNP states and indels were removed. Subsequently, minimum and maximum heterozygosity were set at 40% and 60%. Markers showing significant segregation distortion (χ2 > 12.5, *P* < 0.005, d.f. = 2) were removed. Marker correction and SNP imputing (sliding window = 11) was performed in Tassel and linked markers (distance 150 bp) were binned to obtain 1 SNP marker per locus. The markers were named according to the physical position of the SNP in the reference sequence, e.g. S7_4089911 for marker in chromosome 7 in position 4,089,911.

### QTL analysis

QTL analysis was performed based on symptom scoring data from virus-challenged plants^33^, first by single marker regression, and then by composite interval mapping on genetic maps using an interval of 0.5 cM for mapping SLCCNV and 2 cM for ToLCNDV in QGene^39^. The genetic maps were constructed in JoinMap 4.0 using Kosambi's mapping function with a LOD threshold of 1.00, a rec threshold of 0.4000, a jump threshold of 5.000 and a ripple value of 1. Significance of the marker-trait associations was analyzed by permutation analysis in QGene. The *C. moschata* reference sequence was analyzed for genes in the QTL intervals by manually revising the genes annotated in the QTL region using the genome browser provided by http://cucurbitgenomics.org/JBrowse/.

### Validation

CAPS markers were developed for SNP markers flanking and in the QTL regions of SLCCNV and ToLCNDV using SNP2CASP (http://pgrc.ipk-gatersleben.de/snp2caps/) (Table 1). The markers were applied on a selection of F_1_, F_2_, and BC generation backcrossed to the resistant and the susceptible parent, respectively, using standard PCR and visualization after size separation on 10% polyacrylamide gels and staining with ethidium bromide.

**Table 1:** Markers flanking or located in the QTL intervals associated with SLCCNV or ToLCNDV resistance.

	Marker name	Physical position (bp)	Genetic position (cM)	Forward primer	Reverse primer	Restriction enzyme	Fragment size(s) of the resistant allele (bp)	Fragment size(s) of the susceptible allele (bp)
SLCCNV	S7-3,687,047	Chr. 7 3,687,047	Chr. 07 46.8	TCATTGCAGCAAACCGTAAG	TGCAGTCGACTATCCTGTCG	EcoRI	207 and 81	288
	S7_3,901,603-1	Chr. 7 3,901,603	Chr. 07 51.84	AAATTTGGCTGTCTGGTTGC	TTGGGCTTAGGGCTTAGACA	DraI, MseI	201 and 40	241
	S7_4,017,657	Chr. 7 4,017,657	Chr. 07 52.4	GTGCCACTGAGCTACAAACC	TGAAACGATGGTGGTGAACA	AciI	132 and 19	151
	S7_4,089,911	Chr. 7 4,089,911	Chr. 07 53.5	ACATCCAACCACCTTTCAGC	TCATTAAATCCGTGCCTGCT	BsmAI,Hpy188III	107 and 83	190
ToLCNDV	T8_930,344	Chr. 8 930,344	Chr. 08 8.8	TTGACA TCC CAA GCA AGA TTC CCA T	GAA GGA TGT TGG TGG ACG AG	BccI	126 and 30	156
	T8_937,202	Chr. 8 937,202	Chr. 08 8.9	GCGAACGGAACGTTAGAGAA	TCCTAAAACTTTGGCCGAGA	HphI	204	183 and 21
	T8_115,7451	Chr. 8 1,157,451	Chr. 08 10.7	AAACCGATTGGATCAGGAAA	AACATCTCGGCCAAGTCAAC	Hpy188I	134	91 and 43
	T8_1,203,346	Chr. 8 1,203,346	Chr. 08 11.3	CAGATCAGCCAACTCTAACATGTAAGAATC	ACGTCACAAGGGAGTTGTTCAG	TaqI	172	143 and 29
	T8_1,235,474	Chr. 8 1,235,474	Chr. 08 12.4	ACGACGAGGTGAGACCTGTT	GCGTTCACTATGGTCCGAAT	HpyCH4III	213 and 62	275

## Results

### Resistance of pumpkin line AVPU1426 to squash leaf curl China virus infection

The screening results for SLCCNV resistance in an F_2_ population derived from the cross Waltham Butternut × AVPU1426 were described in^33^. All virus-inoculated plants of the susceptible parent were stunted, with curled and yellowing leaves and reduced leaf lamina (symptom score = 4), while plants of the resistant parent showed only very mild or no virus infection symptoms (symptom score = 0 or 1). All F_1_ plants were susceptible (symptom score = 4), and the segregation between resistant and susceptible plants in the F_2_ generation was 1:3 (χ^2^ 0.053, *p* = 0.817), indicating that resistance was conferred by a single recessive gene^33^. A selection of 82 F_2_ plants including 22 resistant plants (score 0 or 1) and 60 susceptible plants (9, 20 and 31 plants with disease scores of 2, 3 and 4, respectively), together with the susceptible and resistant parents were submitted to genotyping by sequencing. In total 116,114,451 sequencing reads of a length of 151 bp each were obtained, and SNP calling, after imputation, control for segregation distortion and subsequent binning of linked markers, resulted in 1,464 SNPs distributed over all 20 chromosomes. The average distance between two marker bins was 182 kbp, with a large gap of 6 Mbp remaining on chromosome 10.

**Figure 1:**
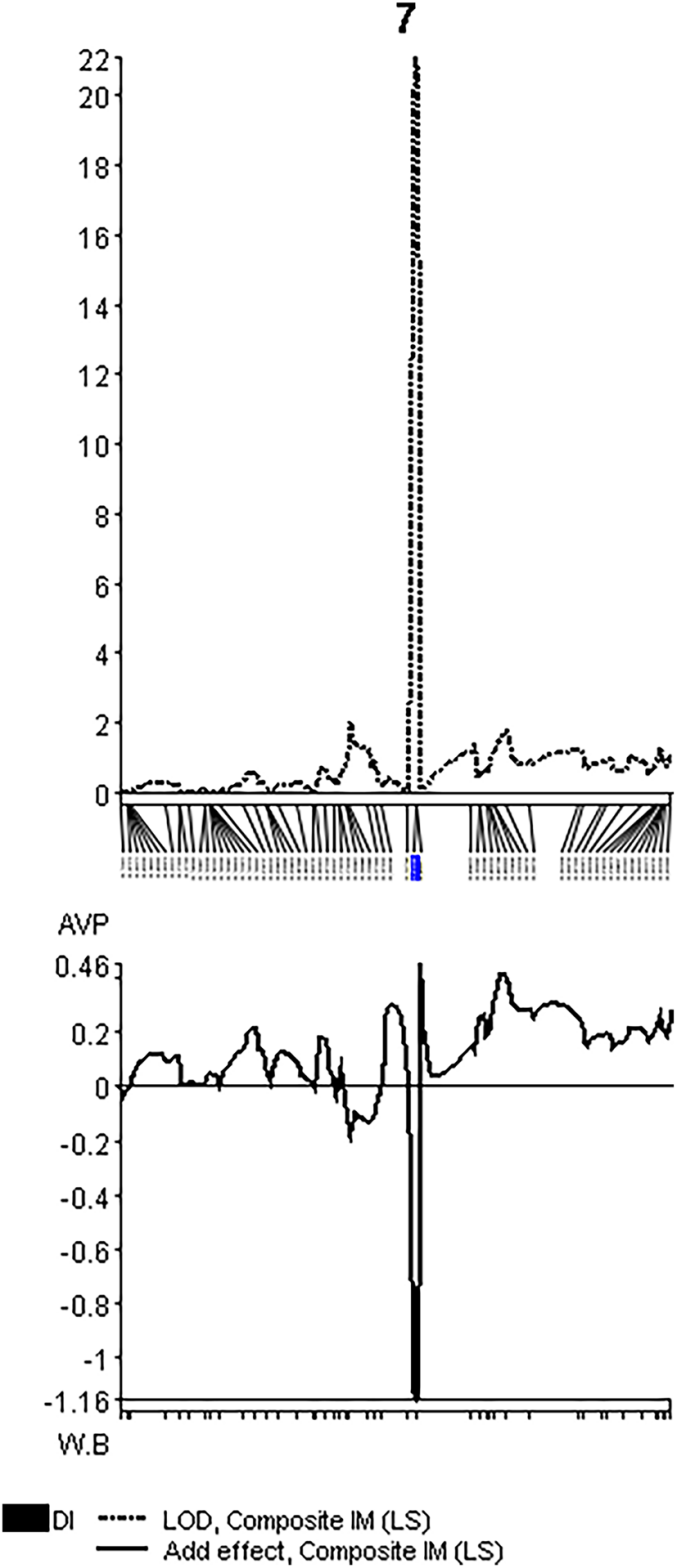
CIM of resistance to SLCCNV in AVPU1426. The resistance is associated with a locus spanning 1.5 cM on chromosome 7.

Single marker regression resulted in three markers located on chromosome 7 between position 39.0 and 41.2 Mbps associated with resistance, with a maximal LOD of 22.9. Composite interval mapping on a genetic map of chromosome 7 showed high association (LOD 19.0 to 23.0) of resistance to a 1.5 cM interval between positions 51.9 and 53.4 cM, corresponding to 188 kb, near marker S7_4,089,911 located on chromosome 7 in position 4,089,911 bp, indicating a single locus contributing to resistance (Fig. 1).

Markers S7_3,687,047, S7_3,901,603-1, S7_4,017,657 and S7_4,089,911 were chosen for validation in 50 BC lines using the susceptible line as recurrent parent (segregation 1:0 susceptible:resistant), 50 BC lines with the resistant line as recurrent parent (segregation in the BC 1:1, 8 F_1_ plants (all susceptible) and 16 F_2_ plants, three of them resistant. This showed that markers S7_4,017,657 and S7_4,089,911 had best prediction accuracy for resistance in these materials compared to the other markers: In 124 tested individuals, these two markers correctly predicted resistance and susceptibility in 122 plants. From one plant no PCR product was obtained, and in one case the marker showed heterozygote genotype predicting susceptibility, while the plant had SLCCNV resistant phenotype (score = 1, Table 2).

**Table 2:** Validation of selected markers located in the QTL interval of SLCCNV resistance in 124 plants. S7-3,687,047 produced 4 wrong predictions, S7_3,901,603-1 produced 2, and use of S7_4,089,911 resulted in 1 false prediction. Resist.: resistant, Suscept.: susceptible.

Marker	S7-3,687,047		S7_3,901,603-1		S7_4,017,657		S7_4,089,911	
Prediction	Resist. (0–1)	Susceptible (2–4)	Resist. (0–1)	Susceptible(2–4)	Resist. (0–1)	Susceptible (2–4)	Resist. (0–1)	Susceptible.(2–4)
RR	19	2	20	1	20	0	20	0
RS	2	71	1	72	1	68	1	75
SS	0	30	0	27	0	34	0	27
Correct prediction	96.80%		98.30%		99.20%		99.20%	
Missing data	0		3		1		1	

The gene content of the QTL region was explored using the *Cucurbita moschata* (Rifu) reference genome sequence and four markers were located in genic regions. Marker S7-3,687,047 was located in an intron region of gene CmoCh07G008020.1 annotated as cellulose synthase (2.4.1.12), and S7_3,901,603-1 was in an intergenic region between CmoCh07G008340.1 (SWIM zinc finger family protein) and CmoCh07G008350.1 (cysteine-rich receptor-like protein kinase). Both markers S7_4,08,9911 and S7_4,017,657 are likely to cause non-synonymous mutations. Marker S7_4,017,657was located in a Syntaxin 121 leading to a conservative change of a valine residue in the susceptible allele to a glycine in the resistant allele. Marker S7_4,089,911 which was nearest to the peak of the QTL collocated with pentatricopeptide repeat proteins (CmoCh07G008660.1), causing an amino acid change from cysteine in the susceptible allele to a phenylalanine in the resistant allele, while the reference sequence of Rifu has a serine residue at this position. Other genes located in the QTL interval included genes such as a E3 ubiquitin-protein ligase RGLG2-like protein gene, and several S-adenosyl-L-methionine-dependent methyltransferases superfamily protein genes.

### Resistance of pumpkin line AVPU1426 to tomato leaf curl New Delhi virus infection

**Figure 2:**
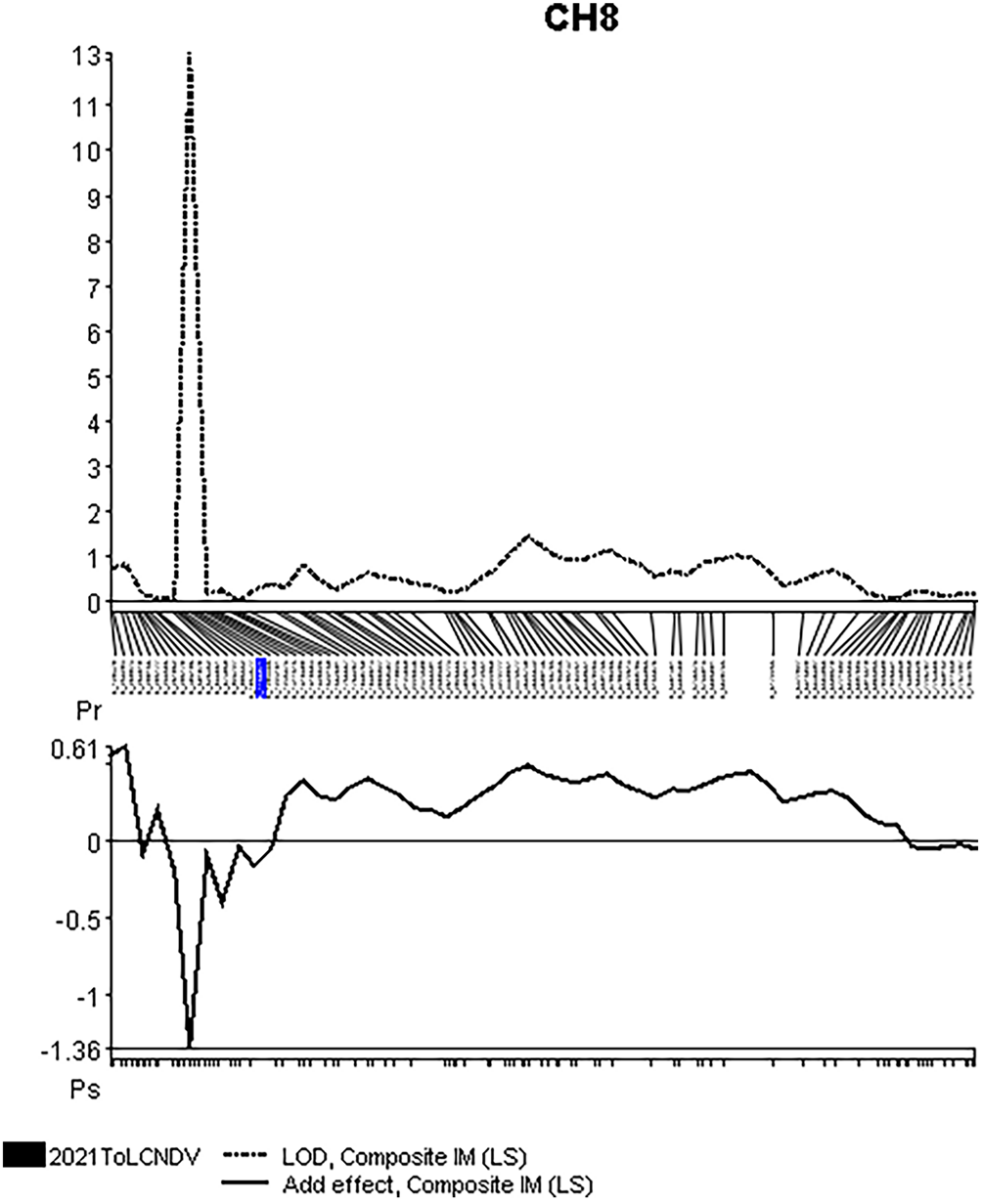
CIM of resistance to ToLCNDV in AVPU1426. The resistance is associated with a locus spanning 1.5 cM on chromosome 8.

The phenotypic analysis of ToLCNDV-challenged pumpkin plants was described by^33^. Segregation in the F_1_, F_2_ and BC generations suggested that resistance relied on a single recessive gene^33^. Paired read sequencing of a genotyping by sequencing library produced from the parental lines and of 84 F_2_ plants segregating for resistance, resulted in 483,388,144 reads, each 2 × 150 bp long. In total, 167,783 raw SNPs were called and after filtering, imputation and binning 2,275 SNPs remained. A genetic map representing all 20 chromosomes was constructed and used for QTL analysis of ToLCNDV resistance. The average distance of markers was 1.16 cM and the map had in total 5 gaps above 10 cM on chromosomes 7, 9, 15 (two gaps) and 18. Single marker regression showed high (LOD > 10) association between 10–10.7 cM and 12.4 and 13.1 cM on chromosome 8. Composite interval mapping suggested a single locus associated with resistance (LOD = 12.2) at position 10.7 cM on chromosome 8 near marker T8_1,053,577 (Fig. 2).

Five markers around the QTL peak spanning 4 cM (or 378.3 kbp) were chosen for validation (Table 1) in 17 F_2_, 39 BC backcrossed to the susceptible parent (all susceptible) and 50 BC backcrossed to the resistant parent (segregation 23:27 resistant:susceptible). Marker T8_1,157,451 had the highest prediction accuracy with 100 out of 106 cases—with 3 false positive predictions, where the resistant allele was detected in plants classified as susceptible, with 2 plants having a disease score of 2 (very light symptoms) and one with severe symptoms (score = 4). Furthermore, this marker suggested 3 false negative predictions, where the heterozygote allele was found in plants classified as resistant (disease score = 0) (Table 3).

**Table 3:** Validation of selected markers located in the QTL interval of ToLCNDV resistance in 106 plants. All tested markers produced at least 3 false results predicting resistance. In two or the 3 cases the plants predicted to be resistant had a relatively low symptom score of 2. Resist.: resistant, Suscept.: susceptible.

Marker	T8-930,344		T8_937,202		T8_1,157,451		T8_1,203,346		T8_1,235,474	
Prediction	Resist.(0–1)	Suscept. (2–4)	Resist. (0–1)	Suscept. (2–4)	Resist. (0–1)	Suscept. (2–4)	Resist. (0–1)	Suscept. (2–4)	Resist. (0–1)	Suscept. (2–4)
RR	24	3	24	3	25	3	25	4	25	4
RS	4	51	4	51	3	50	3	49	3	49
SS	0	24	0	24	0	25	0	25	0	25
Correct prediction	93.40%		93.40%		94.34%		93.40%		93.40%	

For two out of the 5 markers used for validation, the R allele was different from the reference sequence: T8_snp930,344 and T8_1157451. Marker T8930,344 was located in a protein coding region of gene CmoCh08G001610.1 annotated as Chloroplast RNA-binding protein 33. An isoleucine residue in the reference sequence and the susceptible allele was changed to a valine in the resistant allele. T8_1,157,451 was located in gene CmoCh08G001970.1 annotated as an RNA-binding protein, and the DNA sequence polymorphism at the marker position did not predict an amino acid change in the protein.

## Discussion

Chemical control of intracellular pathogens such as plant viruses is not possible—only their vectors can be controlled by pesticides. Intensive pesticide application is hazardous for farmers, consumers and the environment, and insect vectors can develop resistance against pesticides, often making chemical control of virus vectors not practicable. Therefore, use of host resistance to plant viruses is likely to be the most efficient way to control virus diseases, but recombination of begomovirus genomes in their host plants and vectors leads to rapid evolution and the emergence of new begomovirus strains that may overcome the resistance genes used in current varieties^40^. Breeding for virus resistance is an ‘arms race’, where rapid virus evolution requires continuous efforts to identify new host resistance genes for breeding. Integrated approaches combining adding resistance to virus vectors to host resistance to viruses, may generate a second line of defense against virus diseases, thereby making resistance more durable.

Genetic engineering and gene editing approaches to induce virus resistance in plants were successful^41^, but up to now, only a few virus resistant transgenic crops were adopted by farmers, with PRSV resistant papaya being the first released transgenic virus resistant crop that is widely grown, e.g. in Hawaii^42^. But still, the most common pathway to obtaining virus resistant varieties is the use of resistance sources, mostly landraces or crop wild relatives, to breed resistant cultivars. Therefore, the collection and conservation of cucurbit landraces and wild relatives, and access to these biodiverse materials for breeding, are key for sourcing new genes conferring resistances for emerging diseases including viruses.

Virus resistance in cucurbits has predominantly been obtained from landraces for melon and cucumber, and more so from wild species for *Citrullus* and *Cucurbita*^14^*.* Wild relatives of *Cucurbita* including *C. ecuadorensis, C. foetidissima, C. lundelliana, C. ockeechobeensis* and *C. martinezii,* were found to be resistant to a range of viruses including begomovirus^43–45^. Specifically for pumpkin, the African *C. moschata* landrace ‘Nigerian local’ became an important resistance source for breeding. Resistances to ZYMV and PRSV were obtained from this accession, to breed resistant *C. moschata* and *C. pepo* cultivars^46^. Resistance genes to begomovirus were also found in landraces. Resistance to ToLCNDV in two landraces PI 604,506 and PI 381,814 was identified and mapped^16^, and a dominant resistance gene to this virus species was found in *C. moschata* cultivar BSUAL-252^32^. Resistance to multiple begomovirus species in breeding line AVPU1426 was reported by^33^.

The present study mapped two begomovirus resistance genes in AVPU1426, and indentified markers for selecting resistant individuals in breeding. Results also addressed the question of whether the resistance to two different begomovirus species in AVPU1426 relies on a single or two different loci, and whether the resistance gene against ToLCNDV found in AVPU1426 is a new gene, or is likely related to the resistance reported by^16^. It showed that SLCCNV resistance in line AVPU1426 is associated with a locus on chromosome 7, while resistance against ToLCNDV was mapped to chromosome 8, showing that resistance to these two viruses in AVPU1426 relies on two independent loci. While SLCCNV has not been previously mapped in pumpkin, the ToLCNDV resistance locus on chromosome 8 was collocated with a resistance QTL against this virus reported previously. Monogenic recessive inheritance of ToLCNDV resistance was described in the American improved cultivar ‘Large Cheese’ (PI 604,506) and an Indian landrace (PI 381,814), located on top of chromosome 8, at positions 8 and 14 cM, in an interval between physical positions 561,788 and 1,366,729 bp^17^.

The QTL conferring resistance to ToLCNDV in AVPU1426 is located in this interval, suggesting that the resistance locus detected in AVPU1426 is the same as in PI604506 and PI381814, though this has not yet been confirmed by a complementation test that the resistance genes in AVPU1426 and the two resistance sources PI604506 and PI381814 are allelic.

Several markers per locus, including the markers flanking the QTL peak as well as markers located in the peak region, were chosen for validation in F_1_, F_2_ and BC populations. This showed that the best markers predicted the phenotype with high accuracy, albeit not at 100%. The erroneous prediction of SLCCNV susceptibility in one individual that was phenotyped as resistant (score = 1) may have been due to delayed symptom onset or due to a scoring error between symptom scores 1 and 2. The same may have happened with the false predictions of susceptibility for ToLCNDV. During validation of the ToLCNDV markers, the best marker (T8_ 1,157,451) wrongly predicted resistance in plants classified as susceptible in three cases. However, in two of these, the plants had a low symptom score of 2, which classified them as susceptible. These plants may have been resistant, but showed slightly stronger symptoms than the other resistant plants. However, cross over between the marker and resistance gene also could have caused false prediction, e.g. in the case of a ToLCNDV susceptible individual with a symptom score of 4 showing the resistance allele at homozygote state for marker T8_1,157,451. Still, the accuracy of the prediction of resistant plants between 93 and 99% is high enough to recommend markers S7_4,017,657 and S7_4,089,911 for marker-assisted selection of SLCCNV, and T8_1,203,346 for selecting ToLCNDV-resistant *C. moschata* plants.

Parental plants fell into distinct classes and were found to be either resistant (score 0) or susceptible (score 4), while the F_2_ progenies showed a distribution of scores from 0 to 4. This suggests that in addition to the major recessive resistance genes detected in this study, one or more minor genes associated with resistance or susceptibility segregated in the progenies. For example, in *C. melo*, in addition to a major resistance gene to ToLCNDV, two minor resistance genes were found^22^. If such minor resistance genes were present in AVPU1426, they were not detected by QTL mapping. However, high validation rate of the markers for the major resistance genes indicate that that the contribution of the missed minor locus or loci does not strongly affect the resistance phenotype.

Begomoviruses, after infection, reprogram various plant processes to facilitate virus replication and movement, while plants use a combination of transcriptional and post-transcriptional gene silencing as defenses against infection^47^. In addition to transcriptional and post-transcriptional gene silencing, host resistance against begomovirus may include the expression of defense responsive genes and other cellular regulatory genes, while begomovirus pathogenicity is related to the ability of the virus to suppress the host-mediated defense mechanisms^48^. Recessive resistance points towards a process where a viral factor is interacting with a plant function and only a homozygote change in the plant factor, blocks this interaction and leads to resistance. Based on synteny analysis of ToLCNDV loci in *C. moschata* and *C. pepo*,^16^ identified 17 genes conserved in the QTL regions of these two species, including a MAD-box transcription factor CmoCh08G001760 and the transmembrane protein CmoCh08G001780, that showed polymorphisms between ToLCNDV resistant and susceptible plants^49^. A more recent gene expression study found that CmoCh08G001780 is probably silenced through mutations in resistant lines, and further two genes (CmoCh08G001760, CmoCh08G001720) showed moderate changes^50^. The markers identified in the present study are near, but not located in these resistance genes. In *C. melo*, the most likely candidate gene conferring ToLCNDV resistance is MELO3C022319.2^50^ encoding a DNA primase large subunit, which belong to a protein family associated with geminivirus replication^51,52^. The *C. moschata* gene CmoCh08G001720.1 is located in a region that is syntenic to the *C. melo* resistance locus, has an amino acid sequence highly similar to MELO3C022319 and is also annotated as a primase gene (http://cucurbitgenomics.org/). This gene likely acts as a regulatory subunit of primase that generates an RNA primer at the onset of DNA replication of geminiviruses^50^. Deeper molecular studies are required to decipher the roles of the proposed candidate genes in ToLCNDV resistance^53^.

The region where these candidate genes including CmCh08G001720.1 are located, is part of the ToLCNDV resistance QTL of AVPU1426, but the QTL peak of the ToLCNDV resistance QTL in AVPU1426 is located less distal on chromosome 8, at physical position 1,053,577 Mbp around the gene CmCh08G001780.1. Like for the SLCCNV resistance QTL, the gene content of the ToLCNDV resistance QTL was analyzed, and even polymorphisms in genes located in the resistance QTLs were identified. However, without additional research, suggesting candidate genes for resistance remains highly speculative.

## Conclusion

The present study has mapped resistance to SLCCNV and ToLCNDV in the *C. moschata* breeding line AVPU 1426 to chromosome 7 and 8, respectively. The resistance QTL to ToLCNDV is likely the same QTL as reported previously by^16^, while the resistance QTL effective against SLCCNV is newly reported. Validation of the identified markers associated with the QTLs showed a prediction accuracy of 93 to 99% in F_2_ and BC populations derived from AVPU1426, and these are suggested for use in marker assisted breeding to select for virus resistant plants derived from crosses with AVPU1426.

## Data Availability

The generated markers associated with resistance are disclosed in the paper. The sequencing reads generated for population AVPU1426 x Waltham Butternut for mapping SLCCNV are available at https://www.ncbi.nlm.nih.gov/bioproject/PRJNA950483 and the reads for mapping ToLCND are available at https://www.ncbi.nlm.nih.gov/bioproject/PRJNA953224.
